# On the diverse and opposing effects of nutrition on pathogen virulence

**DOI:** 10.1098/rspb.2019.1220

**Published:** 2019-07-10

**Authors:** Victoria L. Pike, Katrina A. Lythgoe, Kayla C. King

**Affiliations:** 1 Department of Zoology, University of Oxford, Oxford OX1 3SZ, UK; 2 Big Data Institute, University of Oxford, Oxford OX3 ZLF, UK

**Keywords:** virulence, nutrition, environmental variation, host–pathogen interactions, immune system, resources

## Abstract

Climate change and anthropogenic activity are currently driving large changes in nutritional availability across ecosystems, with consequences for infectious disease. An increase in host nutrition could lead to more resources for hosts to expend on the immune system or for pathogens to exploit. In this paper, we report a meta-analysis of studies on host–pathogen systems across the tree of life, to examine the impact of host nutritional quality and quantity on pathogen virulence. We did not find broad support across studies for a one-way effect of nutrient availability on pathogen virulence. We thus discuss a hypothesis that there is a balance between the effect of host nutrition on the immune system and on pathogen resources, with the pivot point of the balance differing for vertebrate and invertebrate hosts. Our results suggest that variation in nutrition, caused by natural or anthropogenic factors, can have diverse effects on infectious disease outcomes across species.

## 1. Introduction

Dramatic shifts in global and local environmental conditions are currently being driven by climate change, with major impacts on food security and nutritional availability in ecosystems [1,2]. Additionally, humans have a direct influence on the nutrition of animal and plant populations through, for example, supplementary feeding and fertilizer use [3–6]. In the context of infectious disease, nutrition is hypothesized to be an important factor in infection outcomes by affecting pathogen virulence, which we define broadly here as disease severity caused by an infecting organism (e.g. [7,8]), as well as in other aspects of host–pathogen interactions (e.g. host reproduction [9]). The need to understand the general effects of nutrition on infection outcomes is a pressing issue for species conservation [10], as well as for the study of human disease [11], in a changing world.

**Figure 1.**
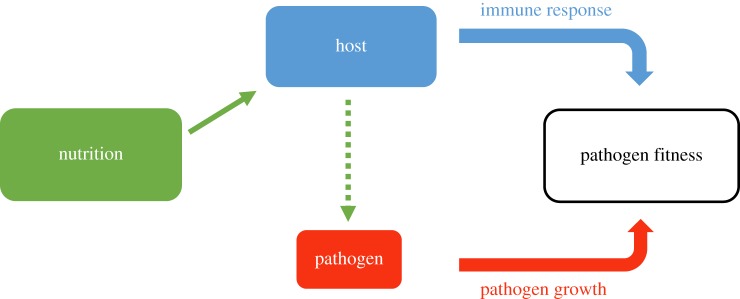
A schematic showing how host nutrition could affect pathogen fitness and disease severity. Nutrition provides energy for the host to use for the immune response and/or directly provides resources for pathogen growth. (Online version in colour.)

Host nutrition can affect infection outcomes by driving separate changes in the host immune system (e.g. [7,12,13]) and pathogen resource availability (e.g. [14,15]) (figure 1). From the host's perspective, it is energetically demanding to maintain an active immune response [16,17]. A decline in their nutritional status could leave hosts less able to suppress infection and, all else being equal, increase the harm caused by the pathogen [7,18]. For example, in domestic canaries (*Serinus canaria*) fed diets supplemented with protein and vitamins, *Plasmodium relictum* exhibited lower parasitemia and virulence (i.e. measured as reduced weight and haematocrit loss) relative to non-supplemented hosts [7]. Alternatively, from the pathogen's perspective, changes in host nutritional quantity (food amount) and quality (specific nutrient content) might affect the availability and type of resources available for pathogen growth during infection [8,19,20]. In this case, the assumption is that faster-growing pathogens, or a higher pathogen fitness/load, would necessarily lead to greater virulence. This assumption, however, is not always valid, such as when hosts are tolerant to the infecting pathogen (e.g. [21] and references therein).

In some systems, there may be other factors which make the impact of nutrition on infection outcomes more complex. For immunopathological diseases, a major contributor of damage to the host is not pathogen load, but the host's own immune system [22]. One hypothesis suggests that providing improved nutrition, and so more energy for the immune system, is thus likely to worsen, not improve, the outcome of these diseases [23]. For pathogens that infect cells of the immune system, such as human immunodeficiency virus (HIV) and its relative simian immunodeficiency virus (SIV), a strong immune response (to HIV, SIV or other pathogens) can result in more targets for infection rather than controlling the infection [24–26]. Beyond the immune system, variation in nutrition can also have an impact on host behaviours. An abundance of food can decrease foraging time, and thus increase the time available for behavioural defences against pathogens, such as grooming [4,27]. Grooming is an effective way of removing ectoparasites [27], such as ticks, which can act as vectors for pathogens such as *Rickettsia* sp.

The energetic demands of the host's immune system might determine the dominant mechanism underlying the link between nutrition and virulence. Given that immune systems are costly to maintain [17,28], reductions in host nutrition could affect the ability of the host to launch an immune response. Despite the large number of similarities between vertebrate and invertebrate immune systems [29], there are also some major differences, such as a lack of acquired immunity in invertebrates [30]. The impact of nutritional changes on the immune system might vary between vertebrates and invertebrates, with the former requiring more resources for an effective immune response. We thus predict that host nutrition will drive changes in virulence via impacts on immune systems in vertebrate host species, but via resource availability to pathogens in invertebrates.

We conducted a meta-analysis to get an overall picture of how nutrition might affect pathogen virulence. We separately considered nutritional quantity (for non-human animals) and quality (for non-human animals, plants and humans). The quantity analysis assessed the changes in pathogen virulence driven by an increase or decrease in host food availability, whereas the quality analysis considered more specific changes to diet that are targeted at the particular host or pathogen, such as variation in vitamins (e.g. [13]) or minerals (e.g. [14]). To understand the mechanism by which nutrition affects pathogen virulence, we also tested for differences between systems with invertebrate or vertebrate hosts. Subsequently, we discuss the studies in more detail to consider the balance between the effects of host nutrition on the strength of the immune system and the resources available to the pathogen. The immune system is highly complex, but here we simply refer to its ‘strength’ or ability to act to reduce pathogen fitness, enabling us to address this broad evolutionary question across host–pathogen relationships. Overall, the meta-analysis does not reveal a consistent effect across systems. Bringing these studies together highlights how natural or anthropogenic changes in nutrient availability might cause diverse effects on virulence across species.

## 2. Meta-analyses

### (a) Literature search

To gather quantitative evidence of the impact of host nutrition on pathogen virulence, we conducted a literature search on Web of Science extracting data from papers up to and including 4 February 2019 (see electronic supplementary material, figure S1 for PRISMA flow chart [31]). We then performed backwards and forwards citation searches on the papers of interest. We contacted authors to obtain unpublished data from relevant studies. We used the following selection criterion for inclusion in the meta-analysis:

(1) The study involved an alteration of host nutrient quality or quantity.
(2) The study measured pathogen virulence, in terms of host survival or mortality, at different nutrition levels.
(3) The study was experimental rather than observational. This restriction allowed us to collate controlled tests of the effects of nutrition.

In total, we found 35 papers that fulfilled these criteria. These papers incorporated a variety of hosts: 32 non-human animal species, two plant species, one unicellular ciliate and three studies on human patients. This enabled us to obtain 52 effect sizes. Of these effect sizes, 33 (from 23 studies) altered the quality of the host diet, and 19 (from 12 studies) altered the quantity of the host diet.

### (b) Statistical analysis

We used Hedges's *g* [32] as our measure of effect size, as it is not biased by small sample sizes [33]. We first determined Cohen's *d*, using the formula below, and later we converted this into Hedges's *g*.

$$\mathrm{Cohe}n^{\prime }s\;\;d=\frac{M_{1}-M_{2}}{S_{\mathrm{pooled}}}\;\;\mathrm{where}\;\;S_{\mathrm{pooled}}=\sqrt{\frac{(n_{1}-1)s_{1}^{2}+\;(n_{2}-1)s_{2}^{2}}{\left(n_{1}-1)+(n_{2}-1\right)}}.$$

Here, *M*_1_ and *M*_2_ refer to the mean virulence resulting from each of the treatment groups, and *s*_1_ and *s*_2_ refer to the standard deviations of the mean virulence for the treatment groups. In all comparisons, *M*_1_ refers to the higher nutrition treatment group, and *M*_2_ to the lower nutrition treatment group. In our analysis, we have three different ‘treatment types’: *high versus control*, *control versus low* and *high versus low*. For the treatments of *high versus control*, *M*_1_ refers to the high treatment group and *M*_2_ to the control group; for *control versus low*, *M*_1_ refers to the control group and *M*_2_ to the low treatment group; and for *high versus low* (studies which compared high and low treatments without a control), *M*_1_ refers to the high treatment group and *M*_2_ to the low treatment group.

We converted Cohen's *d* into Hedges's *g* using the following formula:

$$\mathrm{Hedg}es^{\prime }s\;\;g=J\times \;\mathrm{Cohe}n^{\prime }s\;\;d,$$

where

$$J=\;1-\;\frac{3}{4(n_{1}+n_{2}-2)-1}.$$

Next, we calculated the variance of Hedges's *g* (*V_g_*) using:

$$V_{g}=J^{2}\times \;\mathrm{Cohe}n^{\prime }s\;\;d.$$

Values of Hedge's *g* equal to zero represent no difference in pathogen virulence between treatments. Positive Hedges's *g* values represent cases where the pathogen virulence is greater in the higher nutrition treatment group than the lower nutrition group. Conversely, negative Hedges's *g* values represent cases where the pathogen virulence is greater in the lower nutrition treatment group than the higher nutrition group.

We then split the data into studies according to whether they investigated nutritional quality or quantity. We used R v. 3.4.3 [34] and RStudio v. 1.0.153 [35] for statistical analyses of the quality and quantity data. We constructed multi-level meta-analytic models using the package ‘metafor’ [36]. This multi-level model was required as there were multiple effects sizes from some studies [37]. We included two random effects on the model intercept: (1) a study-level random effect (the level of the model at which variation between studies is distributed), and (2) an observation-level random effect (the level of the model at which variance within studies is distributed) [37]. We used a restricted maximum-likelihood estimation method (REML) for estimating parameters. The overall relationship between host nutrition (quality or quantity) and pathogen virulence could be affected by a number of other variables such as: treatment type (*High versus Control*, *Control versus Low*, *High versus Low*), host type (broad taxonomic groupings and invertebrate versus vertebrate hosts) and pathogen type (broad taxonomic groupings). Thus, we performed univariate moderator analyses to investigate the effects of these additional variables [37]. To determine the significance of these moderator variables we carried out analysis of variance (ANOVA). Any groups with sample sizes lower than three were excluded from the moderator analyses. To test for publication bias, we tested for funnel plot asymmetry and then carried out a trim and fill analysis using the package ‘metafor’ [36]. We also used the packages ‘ggplot2’ [38] and ‘cowplot’ [39] for producing figures and ‘dplyr’ [40] for data manipulation.

In our data, we identified four extreme values that could be considered as outliers, two in the quality data (see electronic supplementary material, table S1) and two in the quantity data (see electronic supplementary material, table S2). To investigate whether or not these outliers were driving our findings, we re-ran all the analyses (outlined above) with the outliers removed.

### (c) Results of the meta-analyses

**Figure 2.**
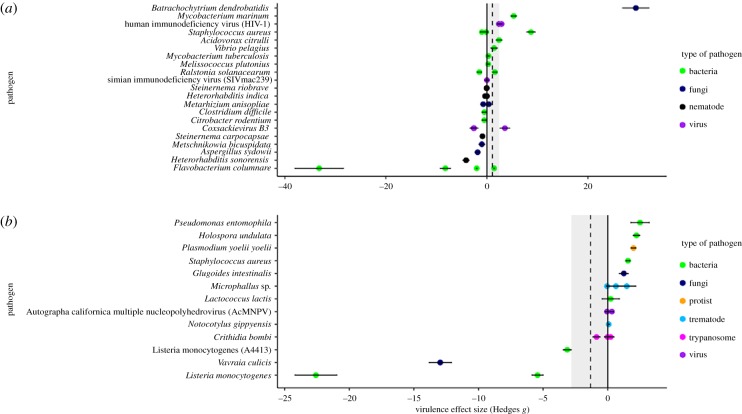
Forest plots of nutrient quality (*a*) and quantity (*b*) and their effects on virulence effect size (Hedges's *g* ± 1 s.e.) for each pathogen species. Dotted line shows the overall effect size with the grey area demarcating the margin of error. Values of Hedges's *g* equal to zero represent no difference in pathogen virulence between treatments. Positive Hedges's *g* values represent cases where the pathogen virulence is greater in the higher nutrition treatment group than the lower nutrition group. Negative Hedges's *g* values represent cases where the pathogen virulence is greater in the lower nutrition treatment group than the higher nutrition group. (Online version in colour.)

**Figure 3.**
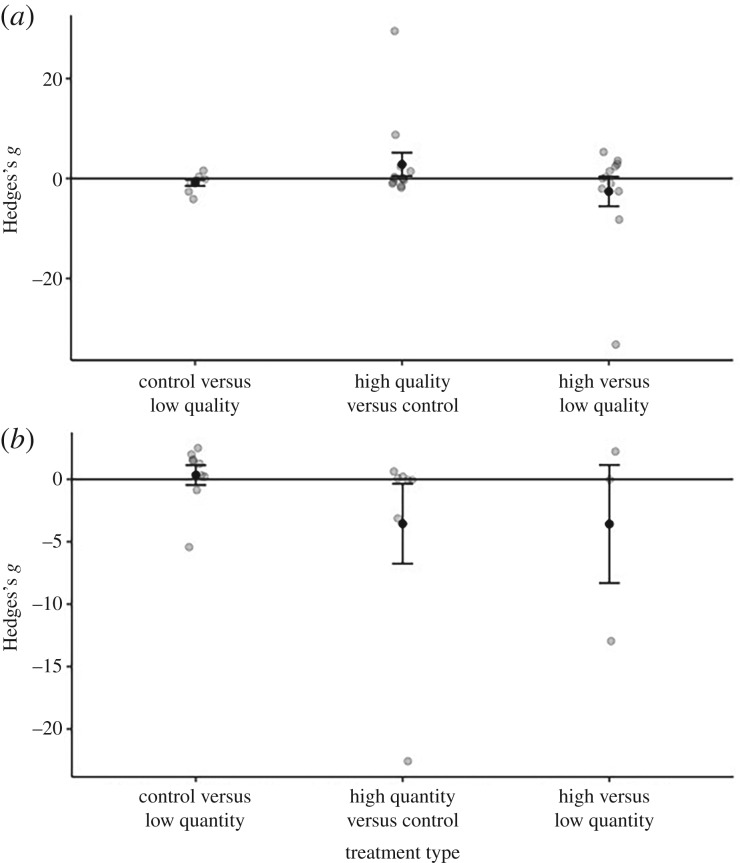
The effect of nutrient quality (*a*) and quantity (*b*) on virulence effect size (Hedges's *g*). Black points represent mean values ± 1 s.e., and raw data are displayed as jittered points.

**Figure 4.**
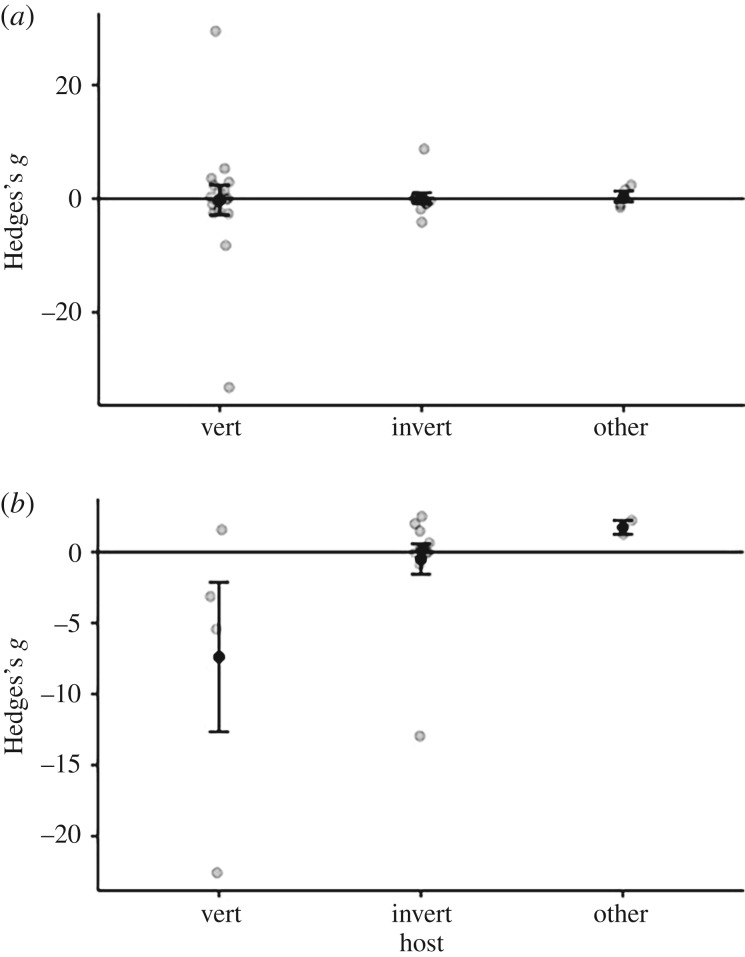
Virulence effect size (Hedges's *g*) for vertebrate and invertebrate hosts as well as ‘other’ hosts (including plants, zooplankton and ciliates) for treatments manipulating nutrient quality (*a*) and quantity (*b*). Black points represent mean values ± 1 s.e. The raw data are displayed as jittered points.

Changes to host nutritional quality or quantity did not have a significant effect on pathogen virulence (figure 2*a*; quality: overall effect size = 1.086 (s.e. = 1.349), *t*_32_ = 0.805, *p* = 0.427, figure 2*b*; quantity: = −1.353 (s.e. = 1.470), *t*_18_ =−0.929, *p* = 0.370). For the quality moderator analyses, we found no significant moderating effects of treatment type (figure 3*a*; high versus control/control versus low/high versus low: *F*_2,30_ = 0.429, *p* = 0.616), host type (electronic supplementary material, figure S2A; figure 4*a*; broad taxonomic grouping: fish/insect/mammal/plant: *F*_3,25_ = 0.317, *p* = 0.813 or invertebrate versus vertebrate: *F*_2,30_ = 0.044, *p* = 0.957) or pathogen type (electronic supplementary material, figure S3A; bacteria/fungi/nematode/virus: *F*_3,29_ = 0.782, *p* = 0.514). Similarly, for the quantity moderator analyses, we found no significant moderating effects of treatment type (figure 3*b*; high versus control/control versus low/high versus low: *F*_2,16_ = 0.877, *p* = 0.435), host type (electronic supplementary material, figure S2B; figure 4*b*; broad taxonomic grouping: insect/mammal/mollusc: *F*_1,14_ = 2.060, *p* = 0.164; or invertebrate versus vertebrate: *F*_2,16_ = 2.646, *p* = 0.102) or pathogen type (electronic supplementary material, figure S3B; broad taxonomic grouping: bacteria/fungi/protist/trematode/trypanosome/virus: *F*_2,11_ = 0.233, *p* = 0.796).

Removing the outliers from our analyses caused changes in individual effect sizes, but crucially did not affect the findings of our study. There was still no overall effect of nutrition on pathogen virulence (see electronic supplementary material, table S3), and the moderator analyses did not produce a significant result (see electronic supplementary material, table S4).

For the quality analysis, there was no evidence for a significant publication bias (see electronic supplementary material, figure S4A; regression test for funnel plot asymmetry: *z* = −1.4822, *p* = 0.1383), but bias was found for the quantity meta-analysis (see electronic supplementary material, figure S4B; regression test for funnel plot asymmetry: *z* = −4.8127, *p* < 0.0001). However, when we removed the outliers from our analyses, we found no publication bias for either quality (see electronic supplementary material, figure S4C; regression test for funnel plot asymmetry: *z* = −0.2857, *p* = 0.7751) or quantity (see electronic supplementary material, figure S4D; regression test for funnel plot asymmetry: *z* = 0.2275, *p* = 0.8200).

## 3. Discussion: nutrition and the balance between host immunity and pathogen resources

We hypothesize that the lack of a significant effect of host nutrition on pathogen virulence is because of the different, and potentially opposing, mechanisms at play. Nutrition can alter both pathogen resources and/or the strength of the host immune response, with these acting to increase and decrease virulence, respectively. We considered the effect of nutrition on pathogen fitness, measured as either as pathogen growth rate or load in a given host environment. Better nutrition might boost the immune system and so reduce pathogen fitness in that environment (e.g. [18]). Conversely, better nutrition might provide more resources to the pathogen, and thus directly increase pathogen fitness (e.g. [8]). Specifically, we hypothesize that the outcome of changes of host nutrition on pathogen virulence depends on the balance between these two effects, with differences in the biology of the host and pathogen systems determining where the equilibrium point lies. For example, energetic requirements of the immune system are likely to be less for invertebrates than for vertebrates, and consequently, nutrition might have less of an effect on invertebrate immunity [30,41].

For those studies used in the meta-analysis that measured immunity and/or pathogen fitness, as well as virulence (rather than just virulence), we broadly determined whether nutrition affected the strength of the immune response based on what was measured. Where possible, we considered the effect of changing host nutrition on both host immunity and pathogen fitness (table 1). For studies where changes in host immunity were not measured, we only considered the effect of nutrition on pathogen fitness (table 2). We discuss these studies in the context of vertebrate and invertebrate hosts.

**Table 1.** Pathogen fitness considering changes to host immune system.

	increase in pathogen fitness	decrease in pathogen fitness	no change
increase in host immunity		coxsackie virus in selenium deficient mice (*Mus* sp.) [13] avian malaria (*Plasmodium relictum*) in canaries (*Serinus canari*) with supplemented diet [7]	
decrease in host immunity	copper deficiency in mice (*Mus* sp.) infected with coxsackie virus B3 [42]	simian immunodeficiency virus in rhesus macaques (*Macaca mulatta*), decline in non-supplemented hosts [43]	
no change	*Staphylococcus aureus* infection in mice (*Mus* sp.) with increased manganese [44]		micronutrient supplementation on humans with HIV [45]

**Table 2.** Pathogen fitness without considering changes to host immune system.

	increase in pathogen fitness	decrease in pathogen fitness	no change
increase in host/pathogen resources	*Metschnikowia bicuspidate* growth in *Daphnia dentifera* with higher quality diet [46] AcMNPV virus production with increased food availability in the cabbage looper (*Trichoplusia ni*) [14] *Daphnia magna* infected with *Pasteuria ramosa* with high quantities of food [47]	*Clostridium difficile* growth in mice (*Mus* sp.) with high-protein diet [48] *Ralstonia solanacearum* in tomato plants (*Lycopersicon esculentum*) with higher calcium [49] HIV patients supplemented with antioxidant micronutrients [50]	
decrease in host/pathogen resources		starved *Bombus impatiens* infected with *Crithidia* trypanosome parasite [51] starved *Daphnia magna* infected with *Glugoides intestinalis* [19]	diet quality on bacterial infection of *Drosophila melanogaster* [52]

Six of the studies considered the host's immune system (table 1), five of which were in mammalian hosts [13,42–45] and one in birds [7]. In all but one of the studies, a decrease in pathogen fitness was observed with an increase in the immune response [7,13], or vice versa [42]. The exception was a study of rhesus macaques (*Macaca mulatta*) infected with SIV [43]. The supplemented macaques experienced both an increase in the number of immune cells and in virus pathogenicity [43], probably because the supplements increased the susceptibility of target (memory CD4^+^ T) cells to SIV infection [43]. Thus, in this SIV study, the nutritional supplements increased resource availability to the virus, outweighing any increase in immune response. The outcome was greater disease severity.

Within these six studies, dietary supplements did not uniformly enhance the immune response (table 1). No effect on immunity was observed when HIV-infected individuals were given microsupplements [45], or when mice infected with the bacteria *Staphylococcus aureus* received manganese supplements [44]. In this latter case, the bacteria in the supplemented hosts had a higher fitness than the un-supplemented hosts because the bacteria was able to use manganese to protect itself against reactive oxygen species and neutrophil killing [44]. Finally, in one case, a deficient diet enhanced the immune response: mice given a selenium deficient diet showed an increase in host immune markers driving down the fitness of the infecting coxsackie virus [13].

Nine of the studies considered how changes to host resources affected pathogen fitness, but did not consider the host's immune system. In these studies, we noted opposite patterns for vertebrate and invertebrate hosts (table 2). For invertebrates—*Daphnia magna* [19,47], *Daphnia dentifera* [46], the cabbage looper (*Trichoplusia ni*) [14] and the bumblebee (*Bombus impatiens*) [51]—improved host nutrition was associated with an increase in pathogen fitness [14,20,47] and reduced host nutrition was associated with a decrease in pathogen fitness [19,51]. No effect on virulence was seen in *Drosophila melanogaster* when its nutrition was decreased [52]. These studies support the hypothesis that an increase in host nutrition can increase host resources available for exploitation by pathogens. By contrast, for mouse [48] and human [50] hosts, an improvement in host nutrition was associated with a decrease in pathogen fitness. This relationship might be indicative of an enhanced host immune response or other defence mechanisms in vertebrates. Finally, for the only plant species considered here, the tomato plant [49], improved host nutrition was associated with decreased pathogen fitness.

## 4. Conclusion

Our study revealed that host nutrition did not have a significant effect on pathogen virulence across systems. We hypothesize that the lack of a general pattern emerges from a balance of both host and pathogen responses to host nutritional status, specifically between impacts on the host immune system (e.g. [7,13]) and resources available to the pathogen [8,19,20].

We predicted there would be differences in the response of the immune system to changes in nutrition for vertebrates compared to invertebrates. Specifically, there may be differences between animal groups in energy requirements for the immune system, with the less ‘complex’ immune system of invertebrates [30,41] requiring fewer resources. In our meta-analysis, we found no significant difference in the effect of dietary quantity or quality on pathogen virulence for vertebrate compared to invertebrate hosts. However, when we looked at a subset of the studies more closely, we found different patterns in vertebrate and invertebrate host organisms (table 1). For the most part, we find that invertebrate hosts display what we would expect under a *pathogen-centric* mechanism—a decrease in fitness with reduced resources for the host, and therefore also the pathogen. Conversely, vertebrate hosts in these studies display what we would expect under a *host-centric* mechanism—a decrease in pathogen fitness with increased resources, due to enhanced host immune responses. Similarly, Cressler *et al.* [15] found increased host nutrition was more likely to lead to an increase in pathogen load for invertebrate, rather than vertebrate, hosts. Further studies are needed to increase the number and types of hosts and pathogens for which we have data, to fully test this hypothesis.

The patterns we discuss here are broad, and on closer inspection there are exceptions. However, importantly, the exceptions we observe make sense. For example, in SIV, where the memory CD4^+^ T are the targets of infection, we observed a decrease in pathogen growth with a decrease in host immunity. Our interpretation of these data may be limited by the fewer studies measuring immune responses (rather than, for example, survival or mortality) in invertebrates in the context of host nutrition. This limitation may highlight a system bias for choosing vertebrate hosts when considering immune function in the context of infection and nutrition. More work is required to study the role of host nutrition on virulence in invertebrate hosts. Moreover, careful consideration of how host nutrition is altered in specific systems is needed. For example, very specific changes to nutritional quality, such as the addition or removal of certain micronutrients (e.g. selenium [13,53]), can have targeted effects on pathogen virulence, which may also help to explain the lack of a directional pattern when examining host nutritional quality and pathogen virulence.

Globally, organisms are facing large changes in nutritional availability [1]. Theoretical work has shown these changes can have far-reaching ecological [54] and epidemiological [55] consequences. For example, the constant supply of resources provided by anthropogenic activities can decrease the propensity of species to migrate and consequently affect infection dynamics within those populations [54]. In addition, resource subsidies can affect the evolution of pathogen virulence and transmission [55]. It is important for us appreciate how widespread the knock-on effects of nutrition induced changes on host–pathogen dynamics and evolution are likely to be. Understanding the long-term consequences of host nutrition on infectious disease will require moving beyond single-generation experiments (as included in this meta-analysis) towards long-term data collection from natural populations (e.g. [56]), evolution experiments (e.g. [57]) or theoretical approaches considering within-host and between-host evolution (e.g. [55,58]).

Understanding the impact of host nutrition on infection outcomes is critical as climate change and human activities are dramatically altering food availability [1–6]. Altering the host diet has a complex relationship with infection, either inhibiting or enhancing disease severity and pathogen proliferation across animal and plant species. We think this complexity precludes a general one-way pattern from emerging across all studies to date. Nonetheless, a greater understanding of the general mechanisms underlying links between nutrition and virulence are needed, with data on both host immunity and pathogen fitness merged. We hypothesize a balance of two factors, the strength of the host immune response and the resources available to the pathogen, shapes the link, but more studies are needed to pin this down. Such efforts will allow us to fully grasp the extent to which the nutritional changes we observe in nature will impact host–pathogen interactions, now and over time.

## Supplementary Material

Supplementary Figures and Tables

## Data accessibility

Data available from the Dryad Digital Repository: https://doi.org/10.5061/dryad.2qt04nm [59]. The code used in this study is available as part of the electronic supplementary material.
