# Supplementary material for: On the diverse and opposing effects of nutrition on pathogen virulence
Source: Proc Biol Sci. 2019 Jul 10;286(1906):20191220. doi: 10.1098/rspb.2019.1220 (PMC6650706; doi:10.1098/rspb.2019.1220)
Supplement: Supplementary Figures and Tables [file rspb20191220supp1.pdf]

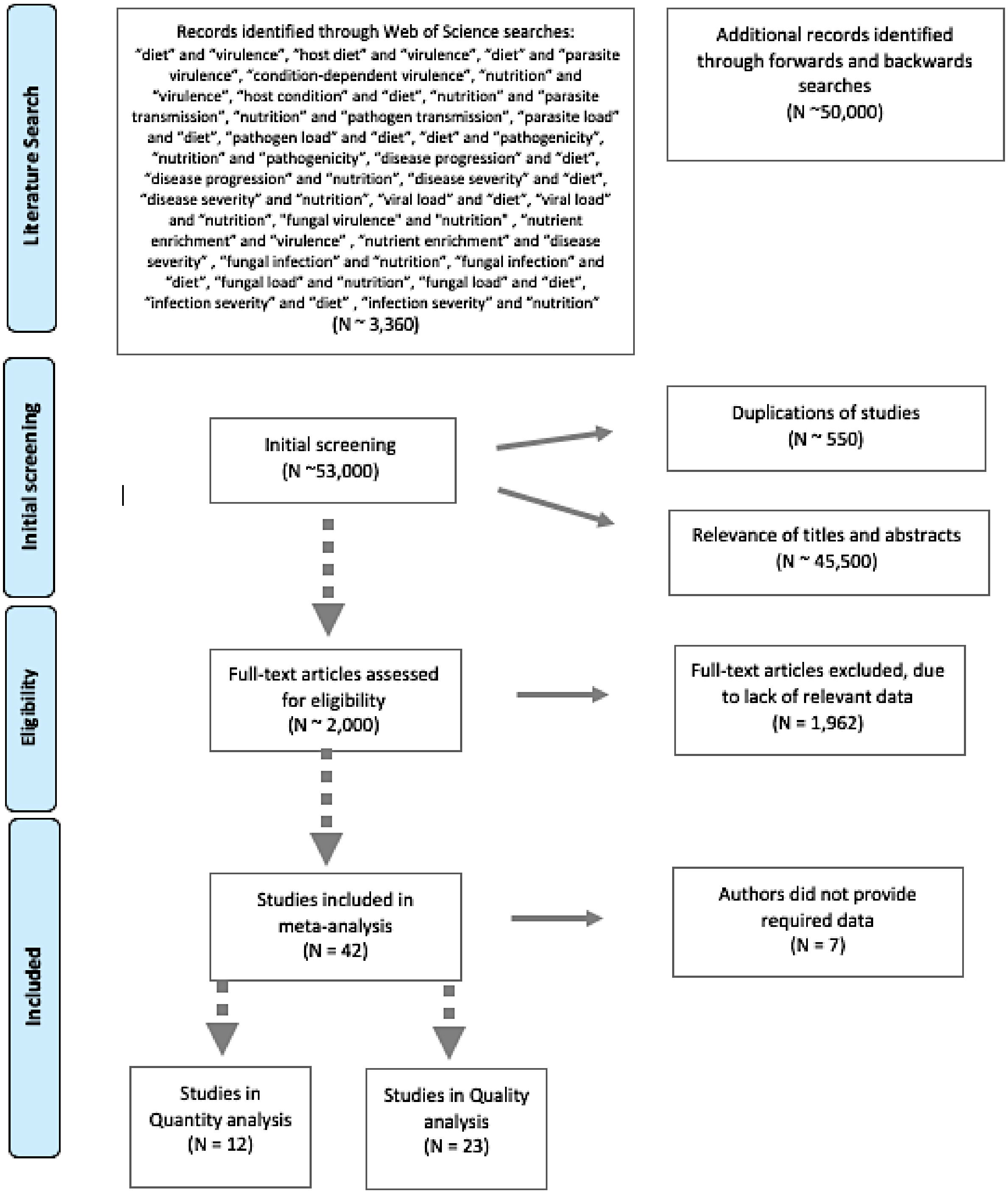

Supplementary Figure 1: PRISMA flow chart detailing data collection.

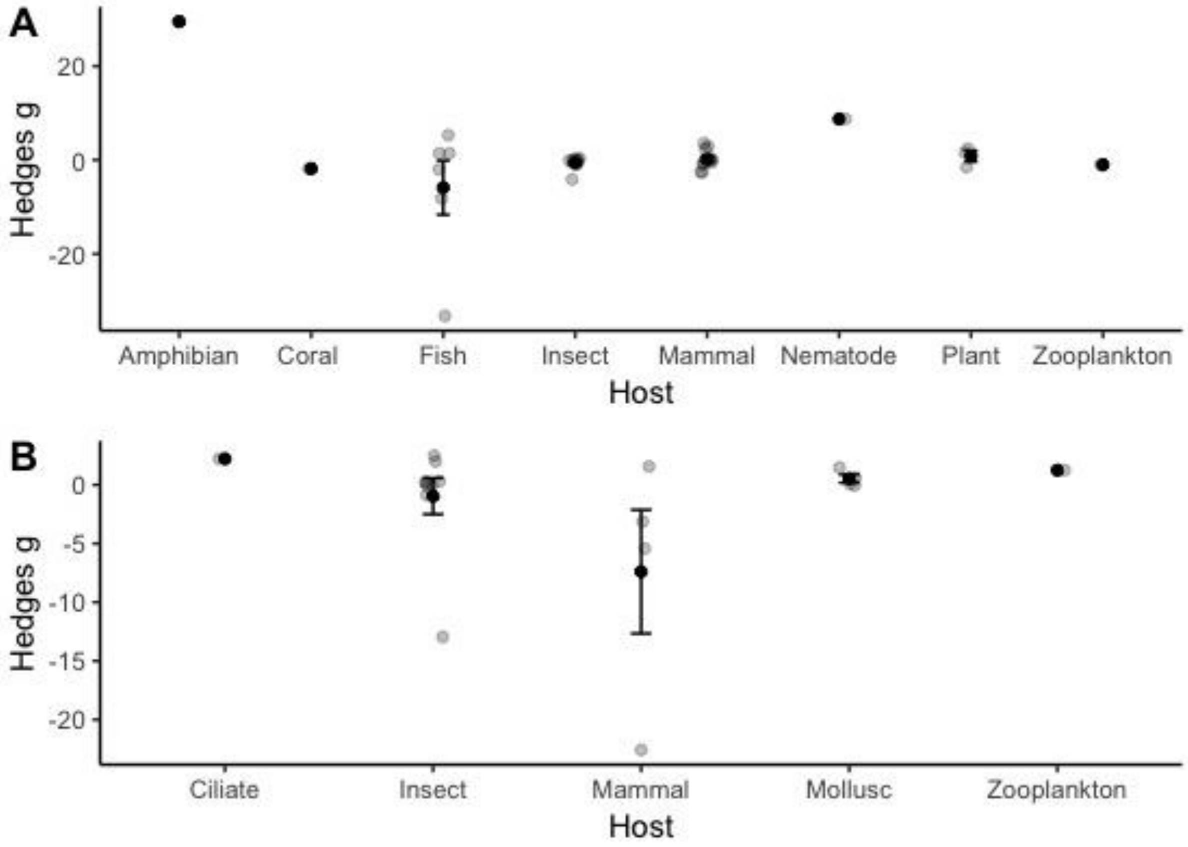

Supplementary Figure 2: Virulence effect size (Hedge's g) for different host types for treatments affecting nutrient quality (A) and quantity (B). Black points represent mean values +/- 1 S.E. Raw data are shown as jittered points.

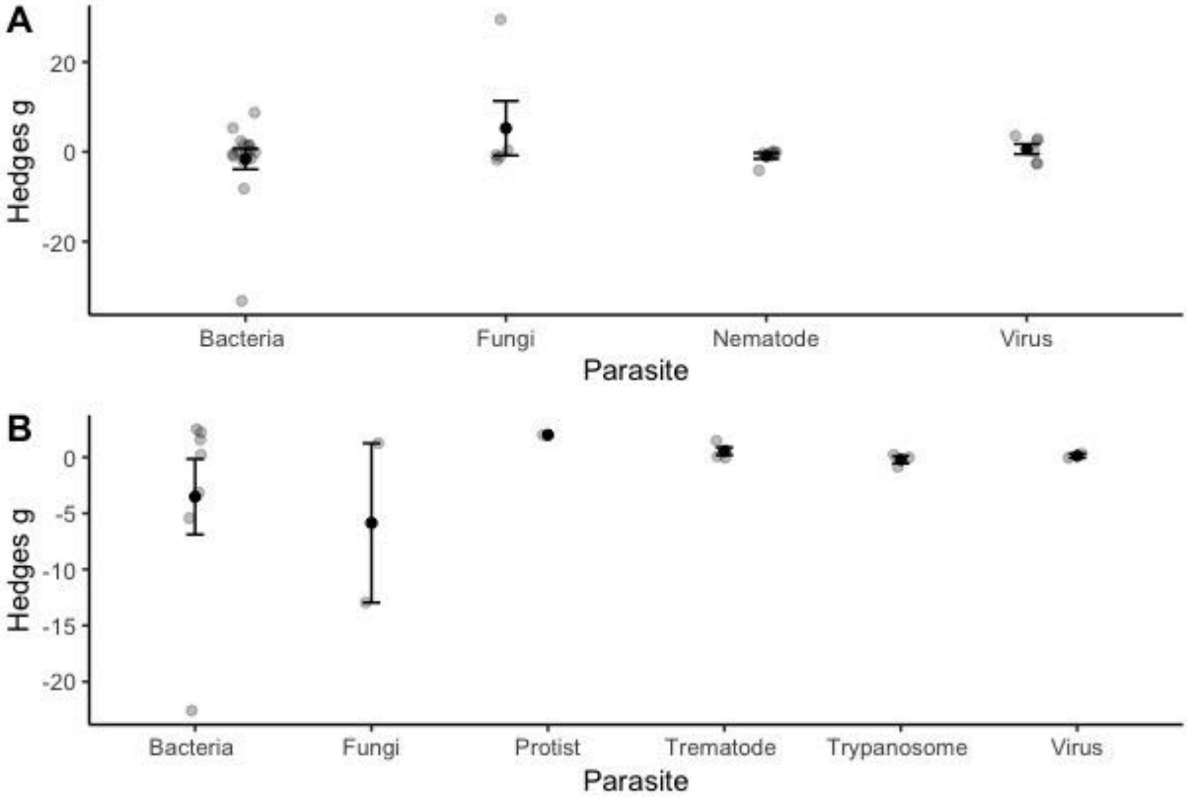

Supplementary Figure 3: Virulence effect size for different pathogen types for treatments affecting nutrient quality (A) and quantity (B). Black points represent mean values +/- 1 S.E.. Raw data are shown as jittered points.

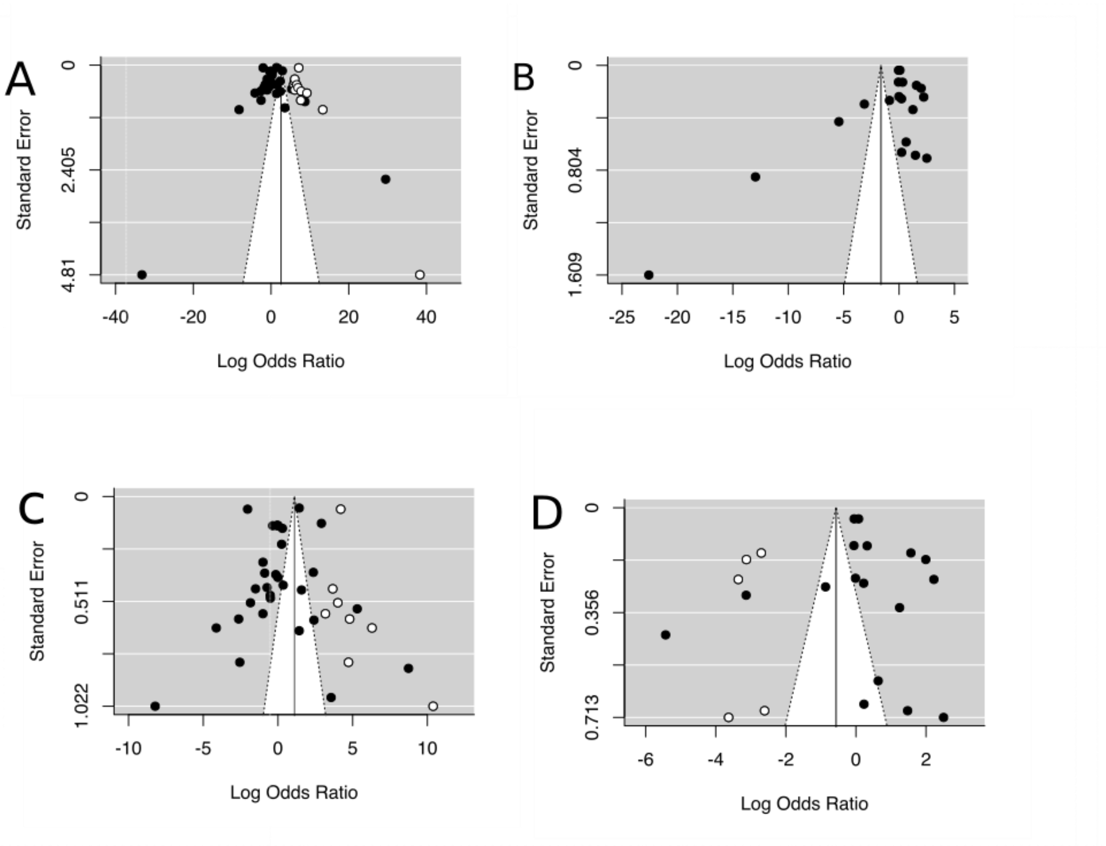

Supplementary Figure 4: Trim and fill plots for the nutrient quality (A, C) and quantity (B, D) meta-analyses, with outliers included (A and B) and removed (C and D).

Supplementary Table 1: A table showing the studies used in the nutrient quality meta-analysis.' * ' denote outliers.

Host (vertebrate (V)/invertebrate (In)/plant (P))	Pathogen	Treatment type	Specific change to nutrition	Change in virulence (+ increase, - decrease, = no change)	Hedge's g	Refs
Caenorhabditis elegans (In)	Staphylococcus aureus (E)	High v Control	Glucose	+	8.739	(64)
Mus sp (V)	Coxsackievirus B3	High v Low	Vitamin E	+	-2.553	(32)
Mus sp (V)	Staphylococcus aureus (E)	High v Control	Manganese	+	3.559	(47)
		Control v Low	Manganese	+	-1.002	
Plutella xylostella (In)	Metarhizium anisopliae (E)	High v Control	Carbon and Nitrogen	-	-0.732	(65)
		Control v Low	Carbon and Nitrogen	-	0.352	
Mus sp (V)	Coxsackievirus B3	Control v Low	Selenium	-	-2.630	(14)
Mus sp (V)	Citrobacter rodentium (E)	Control v Low	Selenium	-	-0.518	(55)
Tenebrio molitor (In)	Heterorhabditis indica (E)	High v Control	Lipid	+	-0.352	(66)
			Protein	-	0	
Tenebrio molitor (In)	Steinernema riobrave (E)	High v Control	Lipid	=	-0.075	(66)

			Protein	=	-4.128	
Manduca sexta (In)	Heterorhabditis sonorensis (E)	Control v Low	Lower quality feed	=	-0.886	(67)
Manduca sexta (In)	Steinernema carpocapsae (E)	Control v Low	Lower quality feed	+	1.424	(67)
Ictalurus punctatus (V)	Flavobacterium columnare (E)	High v Low	Lower $CuSO_4$ supplementation	-	-2.033	(68)
			Higher $CuSO_4$ supplementation	=	0.247	
Apis mellifera (In)	Melissococcus plutonius (E)	High v Low	Potassium	=	0.247	(69)
Daphnia dentifera (In)	Metschnikowia bicuspidate (E)	High v Low	Environmental nutrients	=	-1.007	(50)
Morone saxatilis (V)	Mycobacterium marinum (Int)	High v Low	Lower quality feed	+	5.306	(70)
Macaca mulatta (V)	Simian Immunodeficiency Virus (SIV) (Int)	High v Low	Micronutrient supplements	+	-0.002	(46)
Homo sapien (V)	Mycobacterium tuberculosis (Int)	High v Control	Vitamin A and Zinc	=	0.298	(71)

Homo sapien (V)	Human immunodeficiency virus HIV-1 (Int)	High v Control	Vitamin E and C	-	2.368	(54)
Mus sp (V)	Clostridium difficile (E)	Control v Low	Protein	=	-0.505	(53)
Cucumis melo (P)	Acidovorax citrulli (E)	High v Low	Potassium	-	2.409	(72)
Lycopersicon esculentum (P)	Ralstonia solanacearum (E)	Control v Low	Calcium	-	1.579	(33)
		High v control	Calcium	-	-1.500	
Homo sapien (V)	Human immunodeficiency virus HIV-1 (Int)	High v Low	Micronutrient supplements	-	2.910	(48)
Dicentrarchus labrax (V)	Vibrio pelagius	High v Control	Shrimp protein hydrolysate	-	1.424	(73)
Oncorhynchus mykiss (V)	Flavobacterium columnare	High v Low	Growth medium (Shieh broth)	+	-33.212 *	(74)
Danio rerio (V)	Flavobacterium columnare	High v Low	Growth medium (Shieh broth)	+	-8.219	(74)
Gorgonia ventalina (In)	Aspergillus sydowii	High v Control	Fertilizer	+	-1.839	(75)
Pseudacris regilla (V)	Batrachochytrium dendrobatidis	High v Control	Nitrogen and Phosphorus	-	29.498 *	(76)

Supplementary table 2:	A table showing all of the studies used in the nutrient quantity meta-analysis. ' * ' denote outliers				
Host (Vertebrate (V)/ Invertebrate (In))	Pathogen	Treatment type	Change in virulence (+ increase, - decrease, = no change)	Hedge's g	Study
Mus sp (V)	*Staphylococcus aureus* (E)	Control v Low	+	1.568	(77)
Paramecium caudatum (ciliate)	*Holospora undulata* (Int)	High v Low	+	2.224	(78)
Aedes aegypti (In)	*Vavraia culicis*	High v Low	+	-12.959*	(8)
Potamopyrgus antipodarum (In)	*Microphallus sp* (E)	High v Control	=	-0.051	(79)
Potamopyrgus antipodarum (In)	*Notocotylus gippyensis* (E)	High v Control	+	0.075	(79)
Bombus impatiens (In)	*Crithidia sp* (E)	High v Low	-	-0.013	(51)
Trichoplusia ni (In)	Autographa californica multiple nucleopolyhedrovirus AcMNPV (Int)	Control v Low	+	0.319	(15)
		High v Control	+	-0.058	
Bombus terrestris (In)	*Crithidia bombi*	High v Control	-	0.219	(80)
		Control v Low	-	-0.868	
Mus sp (V)	*Listeria monocytogenes* (Int)	High v Control	-	-3.133	(81)
Cavia porcellus (V)	*Listeria monocytogenes* (Int)	High v Control	-	-22.580*	(81)
		Control v Low	-	-5.431	
Anopheles stephensi (In)	*Plasmodium yoelii yoelii* (Int)	Control v Low	+	1.992	(82)
	Microphallus sp (E)	Control v Low	+	1.474	(83)

Potamopyrgus antipodarum (In)					
		High v Control	+	0.634	
Daphnia magna (In)	*Glugoides intestinalis* (Int)	Control v Low	-	1.245	(20)
Drosophila melanogaster (In)	*Lactococcus lactis* (E)	Control v Low	=	0.229	(52)
	Pseudomonas entomophila (E)	Control v Low	-	2.500	

Supplementary Table 3: Outlier analysis for overall effect size

**Meta-analysis**	**Outliers**	**Overall Effect size (Hedge's g)**	**Standard error**	**K**	**T-value**	**P-value**
Quality	Included	1.086	1.349	33	0.805	0.427
	Removed	0.187	0.575	31	0.325	0.748
Quantity	Included	-1.353	1.470	19	-0.929	0.307
	Removed	0.406	0.546	17	0.743	0.468

Supplementary Table 4: Outlier analysis for moderator variables

**Meta-analysis**	**Moderator**	**Outliers**	**F – Value**	**P – Value**
Quality	Treatment type	Included	0.429	0.616
		Removed	0.632	0.544
	Vertebrate vs Invertebrate	Included	0.044	0.957
		Removed	0.053	0.948
	Host Type (Broad Grouping)	Included	0.317	0.813
		Removed	0.276	0.842
	Pathogen Type (Broad Grouping)	Included	0.782	0.514
		Removed	0.456	0.716
Quantity	Treatment type	Included	2.060	0.164
		Removed	0.249	0.783
	Vertebrate vs Invertebrate	Included	2.646	0.102
		Removed	0.199	0.174
	Host Type (Broad Grouping)	Included	0.233	0.796
		Removed	0.918	0.426
	Pathogen Type (Broad Grouping)	Included	0.233	0.796
		Removed	0.042	0.959

Full reference list including studies used in the meta-analysis (Supplementary Table 1 and 2):

1. Cahill AE, Aiello-Lammens ME, Caitlin Fisher-Reid M, Hua X, Karanewsky CJ, Ryu HY, et al. How does climate change cause extinction? Proc R Soc B Biol Sci. 2013;280(1750).
2. Stevenson TJ, Visser ME, Arnold W, Barrett P, Biello S, Dawson A, et al. Disrupted seasonal biology impacts health, food security and ecosystems. Proc R Soc B Biol Sci . 2015 [cited 2018 Apr 30];282(1817):20151453. Available from: http://dx.doi.org/10.1098/rspb.2015.1453
3. Oro D, Genovart M, Tavecchia G, Fowler MS, Martínez-Abraín A. Ecological and evolutionary implications of food subsidies from humans. Ecol Lett. 2013 Dec 1;16(12):1501–14. Available from: https://doi.org/10.1111/ele.12187
4. Becker DJ, Streicker DG, Altizer S. Linking anthropogenic resources to wildlife-pathogen dynamics: a review and meta-analysis. Lafferty K, editor. Ecol Lett . 2015 May [cited 2018 May 1];18(5):483–95. Available from: http://doi.wiley.com/10.1111/ele.12428
5. Civitello DJ, Allman BE, Morozumi C, Rohr JR. Assessing the direct and indirect effects of food provisioning and nutrient enrichment on wildlife infectious disease dynamics. Philos Trans R Soc B Biol Sci. 2018;373.
6. Altizer S, Becker DJ, Epstein JH, Forbes KM, Gillespie TR, Hall RJ, et al. Food for contagion: synthesis and future directions for studying host-parasite responses to resource shifts in anthropogenic environments. Philos Trans R Soc Lond B Biol Sci . 2018;373(1745):20170102. Available from: http://www.ncbi.nlm.nih.gov/pubmed/29531154

7. Cornet S, Bichet C, Larcombe S, Faivre B, Sorci G. Impact of Host Nutritional Status on Infection Dynamics and parasite Virulence in a Bird-Malaria System. J Anim Ecol. 2013;83:n/a-n/a.
8. Bedhomme S, Agnew P, Sidobre C, Michalakis Y. Virulence reaction norms across a food gradient. Proc R Soc B Biol Sci . 2004;271(1540):739–44. Available from: http://rspb.royalsocietypublishing.org/cgi/doi/10.1098/rspb.2003.2657
9. Lazzaro BP, Little TJ. Immunity in a variable world. Philos Trans R Soc B Biol Sci . 2009;364(1513):15–26. Available from: http://rstb.royalsocietypublishing.org/cgi/doi/10.1098/rstb.2008.0141
10. Wolinska J, King KC. Environment can alter selection in host – parasite interactions. Trends Parasitol. 2009;25(5):236–44.
11. Chapman CA, Gillespie TR, Goldberg TL. Primates and the ecology of their infectious diseases: How will anthropogenic change affect host-parasite interactions? Evol Anthropol. 2005;14(4):134–44.
12. Smith VH, Jones TP, Smith MS. Host nutrition and infectious disease: An ecological view. Front Ecol Environ. 2005;3(5):268–74.
13. Chandra RK. Nutrition, immunity and infection: from basic knowledge of dietary manipulation of immune responses to practical application of ameliorating suffering and improving survival. Proc Natl Acad Sci. 1996;93(25):14304–7.
14. Beck MA, Kolbeck PC, Shi Q, Rohr LH, Morris VC, Levander OA. Increased virulence of a human enterovirus (coxsackievirus b3) in selenium deficient mice. J Infect Dis. 1994;170(2):351–7.
15. Tseng M, Myers JH. The relationship between parasite fitness and host condition in an insect -virus system. PLoS One. 2014;9(9).

16. Cressler CE, Nelson WA, Day T, McCauley E. Disentangling the interaction among host resources, the immune system and pathogens. Ecol Lett . 2014 Mar 1;17(3):284–93. Available from: https://doi.org/10.1111/ele.12229

17. Sheldon BC, Verhulst S. Ecological immunology - costly parasite defenses and trade-offs in evolutionary ecology. Trends Ecol Evol. 1996;11(96):317–21.

18. McKean KA, Yourth CP, Lazzaro BP, Clark AG. The evolutionary costs of immunological maintenance and deployment. BMC Evol Biol. 2008;8(1).

19. Wakelin D. Nature and nurture: Overcoming constraints on immunity. Parasitology. 1989;99(S1):S21–35.

20. Pulkkinen K, Ebert D. Host starvation decreases parasite load and mean host size in experimental populations. Ecology. 2004;85(3):823–33.

21. Hall SR, Simonis JL, Nisbet RM, Tessier AJ, Cáceres CE. Resource Ecology of Virulence in a Planktonic Host-Parasite System: An Explanation Using Dynamic Energy Budgets. Am Nat . 2009;174(2):149–62. Available from: http://www.journals.uchicago.edu/doi/10.1086/600086

22. Medzhitov R, Schneider DS, Soares MP. Disease tolerance as a defense strategy. Science (80-). 2012;335(6071):936–41.

23. Graham AL, Allen JE, Read AF. Evolutionary Causes and Consequences of Immunopathology. Annu Rev Ecol Evol Syst . 2005;36(1):373–97. Available from: http://www.annualreviews.org/doi/10.1146/annurev.ecolsys.36.102003.152622

24. Long GH, Graham AL. Consequences of immunopathology for pathogen virulence evolution and public health: malaria as a case study, 2011,Evolutionary Applications - Wiley Online Library. Evol Appl . 2011;4. Available from: http://onlinelibrary.wiley.com/doi/10.1111/j.1752-

4571.2010.00178.x/full%5Cnpapers2://publication/uuid/11552CE0-0821-4471-8E9C-C16EF20907DF

25. Douek D, Brenchley J, Betts M, Ambrozak D, Hill B, Okamoto Y, et al. HIV preferentially infects HIV-specific CD4+ Tcells. Nature . 2002;417):2–5. Available from: http://www.nature.com/nature/journal/v417/n6884/abs/417095a.html

26. Modjarrad K, Vermund SH. Effect of treating co-infections on HIV-1 viral load: a systematic review. Lancet Infect Dis. 2010;10(7):455–63.

27. Indrati Hood M, Skaar E. Nutritional immunity: transition metals at the pathogen–host interface. Nat Rev Microbiol. 2012;10(8):525–37.

28. Hart BL. Behavioural adaptations to pathogens and parasites - 5 strategies. Neurosci Biobehav Rev. 1990;14(3):273–94.

29. Sorci G, Faivre B. Inflammation and oxidative stress in vertebrate host-parasite systems. Philos Trans R Soc B Biol Sci . 2009;364(1513):71–83. Available from: http://rstb.royalsocietypublishing.org/cgi/doi/10.1098/rstb.2008.0151

30. Loker E., Adema C., Zhang S, Kelper T., Zha. Invertebrate immune systems-not homogonous, not simple, not well understood. Immunol Rev. 2004;198:10–24.

31. Briggs CJ, Hails RS, Barlow ND, Godfray HJC. The dynamics of insect-pathogen interactions. Ecology of infectious diseases in natural populatons. Cambridge: Cambridge University Press; 1995. p. 295–326.

32. Beck MA, Shi Q, Morris VC, Levander OA. Benign coxsackievirus damages heart muscle in iron-loaded vitamin E-deficient mice. Free Radic Biol Med. 2004;

33. Yamazaki H. Relation between Resistance to Bacterial Wilt and calcium nutrition in tomato seedlings. Japan Agric Res Q. 2001;35(3):163–9.

34. Moher D, Liberati A, Tetzlaff J, Altman D, Group TP. Preferred Reporting Items for

Systematic Reviews and Meta-Analyses: The PRISMA Statement. PLoS Med. 2009;6(7).

35. Hedges L V. Distribution Theory for Glass's Estimator of Effect Size and Related Estimators. J Educ Stat . 1981;6(2):107–28. Available from: http://www.jstor.org/stable/1164588

36. Borenstein M. Introduction to meta-analysis. Chichester : John Wiley & Sons; 2009.

37. R Core Team. R: A Language and Environment for Statistical Computing. 2017; Available from: https://www.r-project.org/

38. RStudio Team. RStudio: Integrated Development for R. RStudio. Boston; 2016.

39. Viechtbauer W. Conducting meta-analyses in {R} with the {metafor} package. J Stat Softw. 2010;36(3):1–48.

40. Assink M, Wibbelink CJM. Fitting three-level meta-analytic models in R: A step-by-step tutorial. Quant Methods Psychol . 2016;12(3):154–74. Available from: https://pure.uva.nl/ws/files/26092656/p154.pdf

41. Wickham H. Elegant Graphics for Data Analysis . New York: Springer-Verlag; 2009. Available from: http://ggplot2.org

42. Wilke C. cowplot: Streamlined Plot Theme and Plot Annotations for "ggplot2." 2019.

43. Wickham H, Francois R, Henry L, Muller K. A Grammar of Data Manipulation. 2017;

44. Gillespie and JP, Kanost MR, Trenczek T. Biological Mediators of Insect Immunity. Annu Rev Entomol . 1997;42(1):611–43. Available from: http://www.annualreviews.org/doi/10.1146/annurev.ento.42.1.611

45. Smith AD, Botero S, Levander OA. Copper deficiency increases the virulence of amyocarditic and myocarditic strains of coxsackievirus B3 in mice. J Nutr . 2008;138(5):849–55. Available from:

http://www.ncbi.nlm.nih.gov/pubmed/18424590%5Cnhttp://jn.nutrition.org/content/138/5/849.full.pdf

46. Goldin BR, Li WJ, Mansfield K, Woods M, Wanke C, Freeman L, et al. The effect of micronutrient supplementation on disease progression and death in simian immunodeficiency virus-infected juvenile male rhesus macaques. J Infect Dis. 2005;192(2):311–8.

47. Juttukonda LJ, Berends ETM, Zackular JP, Moore JL, Stier MT, Zhang Y, et al. Dietary Manganese Promotes Staphylococcal Infection of the Heart. Cell Host Microbe. 2017;22(4):531–542.e8.

48. Jiamton S, Pepin J, Suttent R, Filteau S, Mahakkanukrauh B, Hanshaoworakul W, et al. A randomized trial of the impact of multiple micronutrient supplementation on mortality among HIV-infected individuals living in Bangkok. Aids. 2003;17(17):2461–9.

49. Vale PF, Choisy M, Little TJ. Host nutrition alters the variance in parasite transmission potential. Biol Lett . 2013;9(2):20121145–20121145. Available from: http://rsbl.royalsocietypublishing.org/cgi/doi/10.1098/rsbl.2012.1145

50. Hall SR, Knight CJ, Becker CR, Duffy MA, Tessier AJ, Cáceres CE. Quality matters: Resource quality for hosts and the timing of epidemics. Ecol Lett. 2009;12(2):118–28.

51. Conroy TJ, Palmer-Young EC, Irwin RE, Adler LS, C. P, C. BTJ, et al. Food Limitation Affects Parasite Load and Survival of *Bombus impatiens* (Hymenoptera: Apidae) Infected With *Crithidia* (Trypanosomatida: Trypanosomatidae). Environ Entomol . 2016;45(5):1212–9. Available from: https://academic.oup.com/ee/article-lookup/doi/10.1093/ee/nvw099

52. Kutzer MAM, Kurtz J, Armitage SAO. Genotype and diet affect resistance, survival, and fecundity but not fecundity tolerance. J Evol Biol. 2018;31(1):159–71.

53. Moore JH, Pinheiro CCD, Zaenker EI, Bolick DT, Kolling GL, Van Opstal E, et al. Defined nutrient diets alter susceptibility to clostridium difficile associated disease in a murine model. PLoS One. 2015;10(7).

54. Allard JP, Aghdassi E, Chau J, Tam C, Kovacs CM, Salit IE, et al. Effects of vitamin E and C supplementation on oxidative stress and viral load in HIV-infected subjects. AIDS . 1998;12(13):1653–9. Available from: http://www.ncbi.nlm.nih.gov/pubmed/9764785

55. Smith AD, Cheung L, Botero S. Long-term selenium deficiency increases the pathogenicity of a Citrobacter rodentium infection in mice. Biol Trace Elem Res. 2011;144(1–3):965–82.

56. Beck MA, Handy J, Levander OA. Host nutritional status: The neglected virulence factor. Trends Microbiol. 2004;12(9):417–23.

57. Levander OA, Beck MA. Selenium and viral virulence. Br Med Bull. 1999;55(3):528–33.

58. M. BL, J. HR. Consequences of resource supplementation for disease risk in a partially migratory population. Philos Trans R Soc B Biol Sci . 2018 May 5;373(1745):20170095. Available from: https://doi.org/10.1098/rstb.2017.0095

59. L. HJ, E. CC. Resource-driven changes to host population stability alter the evolution of virulence and transmission. Philos Trans R Soc B Biol Sci . 2018 May 5;373(1745):20170087. Available from: https://doi.org/10.1098/rstb.2017.0087

60. Jokela J, Dybdahl MF, Lively CM. The Maintenance of Sex, Clonal Dynamics, and Host-Parasite Coevolution in a Mixed Population of Sexual and Asexual Snails. Am Nat . 2009;174(S1):S43–53. Available from: http://www.journals.uchicago.edu/doi/10.1086/599080

61. Zeller M, Koella JC. The Role of the Environment in the Evolution of Tolerance and Resistance to a Pathogen. Am Nat . 2017;190(3):000–000. Available from:

http://www.journals.uchicago.edu/doi/10.1086/692759

62. Bonneaud C, Giraudeau M, Tardy L, Staley M, Hill G., McGraw K. Rapid antagonistic coevolution in an emerging pathogen and its vertebrate host. Curr Biol. 2018;28(18):2978–83.

63. Coombs D, Gilchrist MA, Ball CL. Evaluating the importance of within- and between-host selection pressures on the evolution of chronic pathogens. Theor Popul Biol. 2007;72(4):576–91.

64. Lavigne JP, Audibert S, Molinari N, O'Callaghan D, Keriel A. Influence of a high-glucose diet on the sensitivity of Caenorhabditis elegans towards Escherichia coli and Staphylococcus aureus strains. Microbes Infect. 2013;15(8–9):540–9.

65. Wu J, Ali S, Huang Z, Shunxiang R, Cai S. Media Composition Influences Growth, Enzyme Activity and Virulence of the Entomopathogen Metarhizium anisopliae (Hypocreales: Clavicipitaceae). Pak J Zool. 2010;42(4):451–9.

66. Shapiro-Ilan D, Guadalupe Rojas M, Morales-Ramos JA, Lewis EE, Tedders WL. Effects of host nutrition on virulence and fitness of entomopathogenic nematodes: Lipid-and protein-based supplements in Tenebrio molitor diets. J Nematol . 2008];40(1). Available from: https://www.ncbi.nlm.nih.gov/pmc/articles/PMC2586524/pdf/13.pdf

67. Miranda VA, Navarro PD, Davidowitz G, Bronstein J, Stock SP. Effect of insect host age and diet on the fitness of the entomopathogenic nematode-bacteria mutualism. Symbiosis . 2013,61(3):145–53. Available from: https://ezproxy-prd.bodleian.ox.ac.uk:7073/content/pdf/10.1007%2Fs13199-013-0266-7.pdf

68. Farmer BD, Beck BH, Mitchell AJ, Rawles SD, Straus D. Dietary copper effects survival of channel catfish challenged with Flavobacterium columnare. Aquac Res. 2017;48:1751–8.

69. Nakamura K, Yamazaki Y, Shiraishi A, Kobayashi S, Harada M, Yoshiyama M, et al. Virulence Differences among Melissococcus plutonius Strains with Different Genetic Backgrounds in Apis mellifera Larvae under an Improved Experimental Condition. Sci Rep. 2016;6.

70. Jacobs JM, Rhodes MR, Baya A, Reimschuessel R, Townsend H, Harrell RM. Influence of nutritional state on the progression and severity of mycobacteriosis in striped bass Morone saxatilis. Dis Aquat Organ. 2009;87(3):183–97.

71. Visser ME, Grewal HM, Swart EC, Dhansay MA, Walzl G, Swanevelder S, Lombard C, Maartens G. The effect of vitamin A and zinc supplementation on treatment outcomes in pulmonary tuberculosis: a randomized controlled trial. Am J Clin Nutr 2011. 2011;93(1):93–100.

72. Zimerman-Lax N, Tamir-Ariel D, Shenker M, Burdman S. Decreased potassium fertilization is associated with increased pathogen growth and disease severity caused by Acidovorax citrulli in melon foliage. J Gen Plant Pathol. 2018;84(1):27–34.

73. Gisbert E, Fournier V, Solovyev M, Skalli A, Andree KB. Diets containing shrimp protein hydrolysates provided protection to European sea bass (Dicentrarchus labrax) affected by a Vibrio pelagius natural infection outbreak. Aquaculture . 2018;495:136–43. Available from: http://www.sciencedirect.com/science/article/pii/S0044848618301698

74. Kinnula H, Mappes J, Valkonen JK, Pulkkinen K, Sundberg L-R. Higher resource level promotes virulence in an environmentally transmitted bacterial fish pathogen. Evol Appl. 2017 Jun;10(5):462–70.

75. Bruno JF, Petes LE, Harvell CD, Hettinger A. Nutrient enrichment can increase the severity of coral diseases. Ecol Lett. 2003 Dec;6(12):1056–61.

76. Buck JC, Rohr JR, Blaustein AR. Effects of nutrient supplementation on host-pathogen dynamics of the amphibian chytrid fungus: a community approach. Freshw Biol . 2016 Jan 1;61(1):110–20. Available from: https://doi.org/10.1111/fwb.12685

77. Nutini L., Berberic N. Effect of diet and strain difference on virulence of staphylococcus aureus for mice. Appl Microbiol . 1965 ;13(4). Available from: http://apps.webofknowledge.com/full_record.do?product=WOS&search_mode=GeneralSearch&qid=14&SID=E3k115aLxBKHxwlF7G8&page=1&doc=1

78. Restif O, Kaltz O. Condition-dependent virulence in a horizontally and vertically transmitted bacterial parasite. Oikos. 2006;114(1):148–58.

79. Jokela J, Lively CM, Taskinen J, Peters AD. Effect of starvation on parasite-induced mortality in a freshwater snail (Potamopyrgus antipodarum). Oecologia. 1999;119(3):320–5.

80. Sadd BM. Food-environment mediates the outcome of specific interactions between a bumblebee and its trypanosome parasite. Evolution (N Y). 2011;65(10):2995–3001.

81. Friedman ME, Kautter DA. Effect of nutrition on the respiratory virulence of Listeria monocytogenes. J Bacteriol. 1962;83(3):456–62.

82. Moller-Jacobs LL, Murdock CC, Thomas MB. Capacity of mosquitoes to transmit malaria depends on larval environment. Parasites and Vectors. 2014;7(1).

83. Krist AC, Jokela J, Wiehn J, Lively CM. Effects of host condition on susceptibility to infection, parasite developmental rate, and parasite transmission in a snail-trematode interaction. J Evol Biol. 2004;17(1):33–40.
